# Rheology, Microstructure, and Storage Stability of Emulsion-Filled Gels Stabilized Solely by Maize Starch Modified with Octenyl Succinylation and Pregelatinization

**DOI:** 10.3390/foods10040837

**Published:** 2021-04-12

**Authors:** Myeongsu Jo, Min Jea Chang, Kelvin K. T. Goh, Choongjin Ban, Young Jin Choi

**Affiliations:** 1 Department of Agricultural Biotechnology, Seoul National University, Seoul 08826, Korea; cmso5517@snu.ac.kr; 2 Center for Food and Bioconvergence, Seoul National University, Seoul 08826, Korea; 3 Dongsuh Foods Corporation, Incheon 21314, Korea; mjjang@dongsuh.co.kr; 4 School of Food and Advanced Technology, Massey University, Palmerston North 4442, New Zealand; K.T.Goh@massey.ac.nz; 5 Department of Environmental Horticulture, University of Seoul, Seoul 02504, Korea; 6 Research Institute of Agriculture and Life Sciences, Seoul National University, Seoul 08826, Korea

**Keywords:** emulsion-filled gels, octenyl succinic anhydride-modified and pregelatinized maize starch, rheological property, microstructure, storage stability

## Abstract

We prepared emulsion-filled gels stabilized using octenyl succinic anhydride-modified and pregelatinized maize starch (OSA-PGS). The effect of the oil volume fraction (Φ, 0.05–0.20) and OSA-PGS concentration (3–10% *w*/*v*) on the rheological and microstructural properties of the emulsion-filled gels was evaluated. Confocal fluorescence images showed that OSA-PGS stabilized the emulsion, indicated by the formation of a thick layer surrounding the oil droplets, and simultaneously gelled the aqueous phase. All of the emulsions exhibited shear-thinning flow behavior, but only those with 10% *w*/*v* OSA-PGS were categorized as Herschel–Bulkley fluids. The rheological behavior of the emulsion-filled gels was significantly affected by both the OSA-PGS concentration and Φ. The mean diameters ($D_{1,0}$, $D_{3,2}$, and $D_{4,3}$) of oil droplets with 10% *w*/*v* OSA-PGS were stable during 30 days of storage under ambient conditions, indicating good stability. These results provide a basis for the design of systems with potential applications within the food industry.

## 1. Introduction

Oil-in-water (O/W) emulsions are commonly used in the food and pharmaceutical industries for protecting lipophilic nutraceuticals from adverse environmental conditions, such as pH, oxygen, and light [1]. Many researchers have recently striven to develop food emulsions stabilized by biopolymers, such as proteins, polysaccharides, and their complexes [2,3]. When biopolymers are applied as emulsion stabilizers, they act as emulsifiers, weighting agents, and reducing coalescence by coating oil droplets [4,5,6]. However, some fluid emulsions are thermodynamically unstable systems that tend to revert to separate water and oil phases over time via coalescence, flocculation, Ostwald ripening, and gravitational separation [7]. The stability of an emulsion can be improved by confining emulsified oil droplets within a polymer gel matrix [8]. This composite system is representative of emulsion-filled gels, which have the advantages of both gels (such as thermodynamic stability) and emulsions (including the ability to load lipophilic nutraceuticals) [9].

As a novel type of structured emulsion, emulsion-filled gels have recently been applied to improve the structure of food products, develop low-fat foods, and encapsulate nutraceuticals [9,10,11]. Fats play an important role in food processing; however, they also contain high amounts of saturated fatty acids, which have adverse effects in several health conditions, such as obesity, cancer, and cardiovascular diseases [12]. Consequently, the food industry is actively seeking alternatives to replace, at least partially, saturated and trans fats in products, such as dairy and processed meats, while preserving their original organoleptic attributes as much as possible to ensure consumer acceptance [4]. The use of synthetic polymers such as polyvinyl alcohol and polysiloxanes as oil structuring agents has been reported, but due to their synthetic nature, these compounds are not accepted as edible matrices [13]. As an alternative, natural compounds such as proteins [3,14,15,16,17] and polysaccharides [4,18,19] are being studied as gelling agents for food-grade emulsion-filled gels. However, mimicking the rheological and textural properties of solid fats using emulsion-filled gels is not trivial, and many factors, such as the physicochemical properties of the gelling matrix, interactions between matrix and oil phases, and oil volume fraction, have an impact on the textural and rheological properties of the product [20,21,22,23]. Therefore, the properties of emulsion-filled gels can be optimized for the selection of appropriate gelling matrices and fillers and by using a rational structural design for the produced materials.

The conventional procedure for producing emulsion-filled gels consists of two steps [15]. The first step is the preparation of an O/W emulsion. During the high-shear mixing procedure, surfactants stabilize the oil droplets electrostatically and/or sterically [24]. The second step involves the formation of a 3D network using heat, gelling agents, salt, and/or acid to entrap the emulsified oil droplets by gelling the aqueous phase [6,16]. This two-step process requires various materials and energy. Additionally, the inhomogeneity of emulsion-filled gel systems is not favorable for large deformation [17]. Therefore, novel and simple methods to replace these complex procedures for preparing emulsion-filled gels are needed.

Starch is a good candidate to stabilize emulsion-filled gels. Octenyl succinic anhydride (OSA)-modified starches are frequently used as food emulsifiers, since they are generally recognized as safe (GRAS) for food use, and the upper limiting level of OSA is 3% per starch weight [25]. Among the various forms of starch typically used as thickeners, pregelatinized starches have an advantage over normal starches in terms of rapid swelling in the absence of heat treatment [26]. Because of this, using pregelatinized starches can reduce the time, energy, labor, and equipment costs of gel production and is appropriate for processing heat-sensitive products. By simultaneously applying the two modifications described above, i.e., OSA modification and pregelatinization, starch becomes a suitable material for simplifying the manufacturing of emulsion-filled gels. Studies using OSA-modified and pregelatinized starch in breadmaking to improve specific volume and texture have been reported [27], but, to the best of our knowledge, there have been no reports of emulsion-filled gels prepared using modified starch in the absence of additional emulsifiers or gelling agents.

In this context, the objective of the present work was to investigate the microstructural and rheological properties, and storage stability of emulsion-filled gels stabilized using OSA-modified and pregelatinized maize starch (OSA-PGS). The influence of the concentration of OSA-PGS and the oil volume fraction on the rheological properties of the emulsions were investigated by evaluating their flow and viscoelastic behaviors. Furthermore, to assess their stability during storage, changes in the mean particle size and microstructure of the emulsion-filled gels were monitored for 30 days. These results provide a basis for the formulation of emulsion-filled gels with applicability to the food industry.

## 2. Materials and Methods

### 2.1. Materials

Maize starch was provided by Tureban (Goyang, Korea). Nile red, OSA, and tricaprylin were purchased from Sigma Aldrich Co. (St. Louis, MO, USA). Ethanol was obtained from Daejung Co. (Siheung, Korea). Concanavalin A conjugated with fluorescein isothiocyanate (Con A) was purchased from VECTOR Laboratories (Burlingame, CA, USA). All chemicals were of analytical reagent grade.

### 2.2. OSA-PGS Preparation

Maize starch (27 g) was dispersed in 900 mL of double-distilled water (DDW) and heated at 105 °C for 15 min in an autoclave (AC-60; Hanyang Scientific Equipment Co., Seoul, Korea) to gelatinize the starch. The resulting starch paste had been slowly cooled to 25 °C. After the pH of the paste was adjusted to 7.5–8.5 with 0.5 M NaOH, 4.05 mL of the OSA solution (0.2 g mL^−1^ in absolute ethanol) was added slowly over 2 h. The reaction between gelatinized starches and OSA was facilitated by stirring for 2 h at pH 8.0–9.0. The pH was adjusted using 0.5 M NaOH. Next, the pH of the suspension was adjusted to 6.5 using 0.5 M sulfuric acid, and the suspension was subsequently mixed with 1800 mL of absolute ethanol to precipitate the OSA-modified starch. The mixture was centrifuged (Supra 22K, Hanil Science Industrial Co., Ltd., Incheon, Korea) at 4000 relative centrifugal force (RCF) for 30 min at 25 °C, and the precipitate was washed twice with ethanol and lyophilized using an FD5508 freeze-dryer (IlShinBioBase Co. Ltd., Yangju, Korea). The lyophilized samples were milled to a powder to obtain OSA-PGS.

As the control sample, pregelatinized maize starch (PGS) was prepared by gelatinizing maize starch (3% *w*/*v*) in an autoclave at 105 °C for 15 min and adding it to 1800 mL of ethanol. The mixture was centrifuged at 4000 RCF for 30 min, and the precipitate was washed twice with DDW and lyophilized.

### 2.3. Determination of the Degree of Substitution for OSA-PGS

The degree of substitution refers to the average number of hydroxyl groups substituted per D-anhydroglucose unit in starch (maximum degree of substitution value, 3.0). The degree of substitution of the prepared OSA-PGS was determined using a previously reported method with slight modification [28]. In brief, OSA-PGS (0.1 g) was dissolved in 10 mL of dimethylsulfoxide by heating (50 °C, 20 min). After cooling to 25 °C, five drops of phenolphthalein indicator were added to the OSA-PGS solution, which was titrated with 0.005 M NaOH until a permanent pale pink color was observed. The degree of substitution was calculated using the following equation [28]:

(1) $$\mathrm{Degree}\;\mathrm{of}\;\mathrm{substitution}=\frac{162\times V\times M}{1000W-210\times V\times M}$$

where V is the volume of 0.005 M NaOH used during titration, M is the molarity of the NaOH solution (0.005 M), and W is the weight of OSA-PGS (0.1 g).

### 2.4. Fourier Transform Infrared Spectroscopy Measurement

Maize starch, PGS, and OSA-PGS were analyzed by a Fourier transform infrared spectrophotometer (Nicolet 6700; Thermo Fisher Scientific, Waltham, MA, USA) at 25 °C to verify esterification between the starch hydroxyl groups and OSA molecules. For each sample, at least 32 scans were performed from 650 to 4000 cm^−1^ at 8 cm^−1^ resolution.

### 2.5. X-ray Diffractometry

The crystal structures of maize starch, PGS, and OSA-PGS powders were investigated using an X-ray diffractometer (D8 Advance; Bruker, Karlsruhe, Germany). Signals at a reflection angle (2*θ*) of 4° to 40° were recorded under Cu Kα radiation (λ = 1.542 Å). The scan speed was 2 steps s^−1^, and the Phi rotation was 30 min^−1^.

### 2.6. Differential Scanning Calorimetry

The thermal properties of maize starch, PGS, and OSA-PGS were determined using a differential scanning calorimeter (Q200; TA Instruments, New Castle, DE, USA). Each sample was dispersed in DDW and placed in a hermetic aluminum pan (sample concentration, 20% *w*/*v*; weight, 10–20 mg) with an empty pan used as a reference. The scans began at 25 °C and increased to 95 °C at a rate of 3 °C min^−1^.

### 2.7. Preparation of Oil-in-Water (O/W) Emulsions Stabilized by OSA-PGS

The O/W emulsions were prepared at 25 °C by mixing tricaprylin with an aqueous phase containing 3, 5, or 10% *w*/*v* OSA-PGS and 0.02% *w*/*v* sodium azide using a high-shear blender (Ultra-Turrax T25D; Ika Werke GmbH & Co., Staufen, Germany) at 15,000 rpm for 2 min. To prepare emulsions, the oil volume fraction (Φ) of each emulsion system was set to 0.05, 0.10, or 0.20, resulting in aqueous volume fractions of 0.95, 0.90, and 0.80, respectively. The emulsions were stored in screw-lid-sealed vials under ambient conditions.

### 2.8. Rheological Characterization 

The rheological properties of the emulsions were measured at 25 °C using a rotational rheometer (RheoStress RS 1; HAAKE Instruments, Karlsruhe, Germany) equipped with a probe with cone-and-plate geometry (diameter, 35 mm; angle, 1°; gap, 52 μm). Prior to the measurements, the sample was left undisturbed on the plate for 5 min to regain its structure and equilibrate to the test temperature. To assess the flow behavior of the emulsions, flow plots of shear stress (τ) versus shear rate ($\dot{\gamma }$) were obtained using a steady-state shear ramp, with $\dot{\gamma }$ ranging from 1.0 to 500.0 s^−1^. Using the subsequent plots, the data were fitted based on the Herschel–Bulkley model: $\tau =\tau_{0}+K{\dot{\gamma }}^{n}$ ($\tau_{0}$, yield stress; K, consistency index; n, flow behavior index).

For dynamic shear measurements, oscillation frequency sweep tests were conducted at frequencies (ω) ranging from 0.1 to 26.8 rad s^−1^, and a constant value of τ (0.6 Pa) was applied to maintain the linear viscoelasticity of the emulsions during the test. The storage modulus ($G^{\prime }$), loss modulus ($G^{\prime \prime }$), and tanδ ($G^{\prime \prime }/G^{\prime }$) were obtained as a function of ω.

### 2.9. Microscopic Observation

The microstructures of emulsions were used to validate the coverage of OSA-PGS on the surface of the oil droplets using a confocal laser scanning microscopy (CLSM; TCS SP8 X; Leica, Mannheim, Germany) with a confocal objective (HC PLAPO 40×/1.4 Oil CS2) and a supercontinuum fiber laser (470–670 nm continuous white-light laser). Emulsions were produced by the homogenizing method. In a 50 mL beaker, 0.5 g of oil phase (0.495 g tricaprylin stained with 0.005 g Nile red) and 9.5 g of aqueous phase (0.3, 0.5, or 1 g OSA-PGS dispersed in 0.02 wt% sodium azide solution) were homogenized at 5000 rpm for 30 s and then incubated with Con A solution (250 mg L^–1^, 100 μL) for 30 min, with stirring, to stain OSA-PGS. A 633 nm laser was used to excite Nile red, and the fluorescence emitted was detected at 650–700 nm. A 488 nm laser was used to excite Con A, and the fluorescence emitted was detected at 500–550 nm. A drop of emulsion was placed on a glass slide, covered with a coverslip, and mounted with oil immersion. The CLSM image was analyzed using Leica Application Suite X software.

The microstructures of emulsions stored for 1 or 30 days at 25 °C were observed using CLSM with confocal scan objectives and a supercontinuum fiber laser. A 633 nm laser was used to excite Nile red, and the fluorescence emitted was detected at 650–700 nm. A drop of emulsion was placed on a glass slide, covered with a coverslip, and mounted with oil immersion. The CLSM image was analyzed using Leica Application Suite X software and used to measure droplet size.

### 2.10. Measurement of Oil Droplet Diameters

The diameters of at least 800 oil droplets (>950 nm; the resolution limit (a pixel area) of CLSM images) in the emulsion systems were determined from CLSM images by image particle analysis using ImageJ (NIH, Bethesda, MD, USA). A known size was set using a scale bar, and based on this, the size of a circular droplet was measured. The images were processed with the color threshold set at default red and dark backgrounds to detect Nile red-labeled oil droplets. After the threshold had been selected, the “analyze particles” settings were set to a pixel size of 0–infinity and circularity of 0.80–1.00 to select only circular droplets, excluding inapposite particles due to overlapping, bleaching, or undyeing. The oil droplets were assumed to be spherical. The number length mean diameter ($D_{1,0}$), surface area moment mean diameter ($D_{3,2}$), and volume moment mean diameter ($D_{4,3}$) were calculated using the following equations:

(2) $$D_{1,0}=\overset{k}{\underset{i=1}{\sum }}\left(N_{i}\cdot D_{i}\right)/\overset{k}{\underset{i=1}{\sum }}N_{i}$$

(3) $$D_{3,2}=\overset{k}{\underset{i=1}{\sum }}\left(N_{i}\cdot D_{i}{}^{3}\right)/\overset{k}{\underset{i=1}{\sum }}\left(N_{i}\cdot D_{i}{}^{2}\right)$$

(4) $$D_{4,3}=\overset{k}{\underset{i=1}{\sum }}\left(N_{i}\cdot D_{i}{}^{4}\right)/\overset{k}{\underset{i=1}{\sum }}\left(N_{i}\cdot D_{i}{}^{3}\right)$$

where D is the diameter of the oil droplets and N is the number of droplets with a specific diameter.

The diameter distribution of the oil droplets in the emulsions was determined as the percentage (area (%)) of the pixel area of the droplets with a specific diameter per the total pixel area of all droplets in the CLSM images. The diameter interval for acquiring the distribution was based on the log-scale interval used in the software of the Mastersizer 2000 instrument (Malvern Instruments Ltd., Malvern, UK). The summed area (%) of droplets with diameters in a specific range was expressed as a bar within the interval range on the horizontal axis in the distribution graph.

### 2.11. Statistical Analyses

The data represent the averages of at least three independent experiments or measurements. To determine the Herschel–Bulkley model parameters ($\tau_{0}$, k, and n), dynamic shear parameters ($k^{\prime }$, $k^{\prime \prime }$, $n^{\prime }$, and $n^{\prime \prime }$) and separation trend in the diameter distribution, regression curves were generated using SigmaPlot 10.0 software for Windows (Systat Software, San Jose, CA, USA). Among the values for the Herschel–Bulkley model parameters, dynamic shear parameters, and mean diameters ($D_{1,0}$, $D_{3,2}$, and $D_{4,3}$), the significance of differences according to sample composition was estimated by analysis of variance (ANOVA) with Tukey’s honest significant difference post hoc test using SPSS software (ver. 25.0; IBM Corp., Armonk, NY, USA). Additionally, the significance of the difference in mean diameter before and after storage was determined using Student’s *t*-test.

## 3. Results and Discussion

### 3.1. Characteristics of OSA-PGS

**Figure 1:**
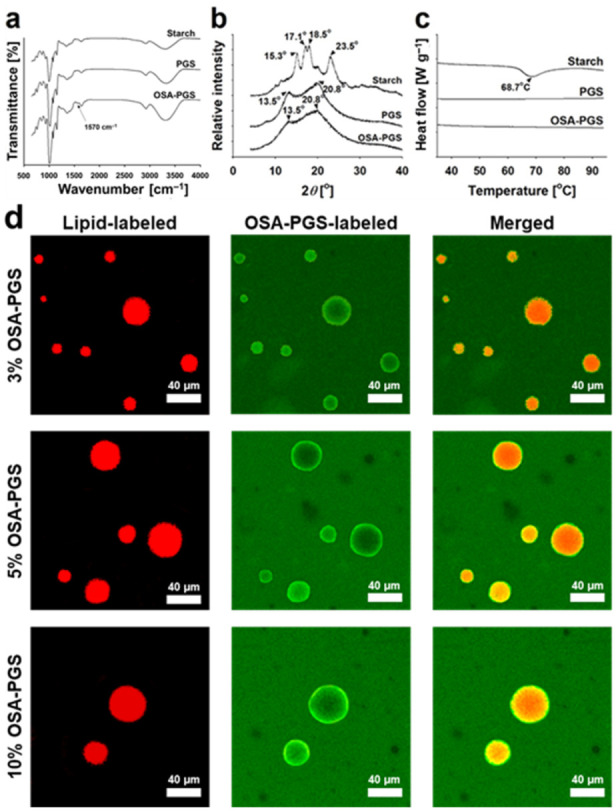
(**a**) Fourier transform infrared spectra, (**b**) X-ray diffractograms, and (**c**) heating calorigrams of maize starch, pregelatinized maize starch (PGS), and octenyl succinic anhydride-modified PGS (OSA-PGS). (**d**) Confocal laser scanning micrographs of emulsions stabilized by 3, 5, or 10% *w*/*v* OSA-PGS and oil volume fraction = 0.05 (Nile red, lipid-labeled; Concanavalin A conjugated with fluorescein isothiocyanate, OSA-PGS labeled).

In this study, OSA-PGS was prepared from maize starch by pregelatinization followed by OSA modification to increase the hydrophobicity of PGS. The degree of substitution of the fabricated OSA-PGS was ~0.011, which is an acceptable value for use in foods since the US Food and Drug Administration set the level of OSAs to 3 wt% (~0.02 in the degree of substitution) in modified starches. Additionally, the infrared spectra of PGS and OSA-PGS showed that OSA and PGS were covalently linked by interesterification, and that the ester bonds were not reduced during precipitation and freeze-drying (Figure 1a). The OSA-PGS spectrum displayed an additional band at 1570 cm^−1^ that was absent from the PGS spectrum. This band indicated that OSA reacted with the hydroxyl groups of starch and was attributed to interesterifications (RCOO groups) [29,30].

Maize starches have an A-type crystallinity, which is in good agreement with the X-ray diffraction (XRD) pattern (Figure 1b). However, the peaks in the XRD patterns of PGS and OSA-PGS were distinctly different from those of maize starch. The peaks in the XRD patterns of OSA-PGS were identical to those of PGS. Specifically, peaks in the XRD pattern of native maize starch were observed at 3.78, 4.79, 5.18, and 5.78 nm, whereas PGS and OSA-PGS exhibited peaks only at 4.27 and 6.55 nm. This indicated that PGS and OSA-PGS had a V-type crystalline structure [31]. The A-type crystalline structure of native maize starch was composed of packed amylose helices. The V-type crystalline structures of PGS and OSA-PGS consisted of inclusion complexes of amylose and ethanol. When the starch paste was mixed with ethanol, a single-helical V-type crystalline structure of amylose and ethanol was formed [32].

Gelatinization of maize starch occurred at approximately 60–80 °C (Figure 1c). However, for pregelatinized starch, the higher the degree of pregelatinization, the smaller the endothermic peak in DSC thermogram [33]. Heating-calorigrams for PGS and OSA-PGS, no phase transition was induced by heating from 25 to 95 °C (Figure 1c). These results indicated that they were fully pregelatinized. Thus, gelling of PGS and OSA-PGS had the advantage of rapid swelling in the absence of heat treatment.

The OSA-PGS covering effect on the surfaces of tricaprylin droplets was verified by the CLSM image obtained after the preparation of coarse emulsions (Figure 1d). Laser irradiation caused the tricaprylin droplets to fluoresce in red in the presence of the Nile red, and the OSA-PGS to fluoresce in green in the presence of the Concanavalin A conjugated with fluorescein isothiocyanate. Several studies have observed OSA-starch granule-stabilized emulsions using CLSM [34]. In those studies, the starch formed at the interface showed Pickering stabilization, where the amphiphilic starch particles stabilized the oil/water interface. Contrary to these results, the micrograph in Figure 1d shows that OSA-PGS was adsorbed in the form of a thick layer on the surface of oil droplets in the gelatinized state. This showed that oil droplets were stabilized by steric hindrance by the OSA-PGS layer on the surface of the oil droplets. At all three OSA-PGS concentrations, OSA-PGS was more concentrated on the surface of oil droplets than in the aqueous phase. Since OSA-PGS is amphiphilic, when oil droplets were dispersed in the aqueous phase by homogenization, OSA-PGS first adsorbed to the oil/water interface to stabilize the oil droplets, and excess OSA-PGS was dispersed in the aqueous phase. The OSA-PGS present in the aqueous phase gelled the aqueous phase to produce emulsion-filled gels. The difference between the fluorescence intensity of the tricaprylin droplet surface and that of the aqueous phase was greatest when 3% *w*/*v* OSA-PGS was used. This meant that the amount of OSA-PGS in the aqueous phase was higher in 5–10% *w*/*v* OSA-PGS than in 3% *w*/*v* OSA-PGS because the amount of Con A present in the system remained the same regardless of the OSA-PGS concentration. Therefore, the emulsion-filled gel is expected to be highly stable due to steric hindrance by the OSA-PGS layer on the surface of tricaprylin droplets and due to the high viscosity of the aqueous phase of OSA-PGS. This is discussed in detail in the following sections.

In summary, PGS produced by the pregelatinization of maize starch swelled in cold water. In OSA-PGS produced by the covalent linkage of PGS with OSA, the ester bond linkages were retained during ethanol precipitation and lyophilization, and OSA modification had negligible effects on the crystallinity and thermal properties of PGS. In addition, the fabricated OSA-PGS stabilized the emulsion in the form of a film on the surface of the oil droplets and gelled the aqueous phase of the emulsion system.

### 3.2. Rheological Properties of the Emulsions and Emulsion-Filled Gels

The appearance of O/W emulsions (Φ = 0.05–0.20) stabilized by 3–10% *w*/*v* OSA-PGS is shown in (Supplementary Materials Figure S1). For all compositions, the side views of vials left undisturbed after emulsion preparation show that the oil droplets were well dispersed in the aqueous phase, and no phase separation occurred during 1 day of storage under ambient conditions. By contrast, phase separation occurred within 6 h when the emulsion was stabilized using PGS. Recently, several groups reported that nonchemical modified gelatinized starch could stabilize O/W emulsions, but the emulsifying ability of the gelatinized starch was insufficient for its application in the food industry [35]. Likewise, the stability of the PGS-stabilized emulsion is maintained only when PGS is subjected to a process that increases the hydrophobicity of the starch, such as OSA modification. Emulsions with Φ = 0.05–0.10 and 3–5% *w*/*v* OSA-PGS flowed like a fluid when the vials were inverted (Figure S1). By contrast, the side views of vials inverted after emulsion preparation revealed a gel-like appearance for emulsions with Φ = 0.20 (with 3–10% *w*/*v* OSA-PGS) or Φ = 0.05–0.20 (10% *w*/*v* OSA-PGS) (Figure S1). Depending on their effect on gel modulus, emulsified oil droplets in the emulsion-filled gel system were classified as active or inactive fillers, depending on whether the emulsion droplet was bound to the gel matrix [16]. The higher the concentration of active filler, the higher the gel modulus. By contrast, emulsion droplets unbound to the gel matrix, which causes a decrease in gel modulus, have been classified as an inactive filler. OSA-PGS-stabilized oil droplets act as active fillers, whose interfacial OSA-PGS interacts not only with the OSA-PGS on the surface of neighboring emulsion droplets but also with the OSA-PGS gel matrix. With an increased amount of active filler, both emulsion stabilization and aqueous phase gelation were obtained using a smaller amount of OSA-PGS. In an emulsion-filled gel system stabilized by OSA-PGS, no strong electrostatic interaction can occur between the oil droplets and gel matrix because the droplets were emulsified with the same starch used in the gel matrix. The interactions between these two elements are similar to those responsible for gel formation, i.e., hydrogen bonding between starch chains [36].

**Figure 2:**
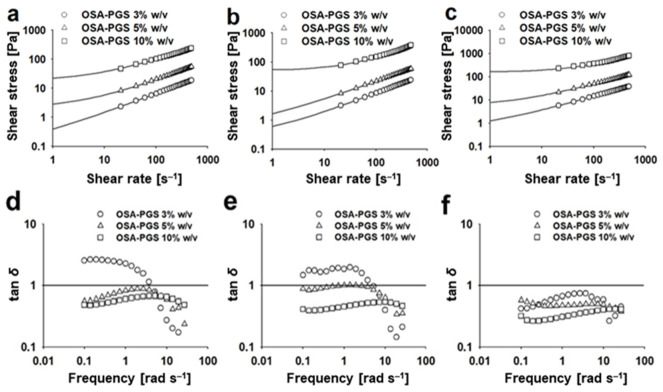
Flow behavior plots (shear stress vs. shear rate) of the emulsions stabilized by pregelatinized maize starch modified with octenyl succinic anhydride (OSA-PGS). Oil volume fraction: (**a**) 0.05, (**b**) 0.10, and (**c**) 0.20. Lines indicate the fitted curves based on the Herschel–Bulkley model ($\tau =\tau_{0}+K{\dot{\gamma }}^{n}$; τ, shear stress; $\dot{\gamma }$, shear rate; $\tau_{0}$, yield stress; K, consistency index; n, flow index). Loss tangent angles ($\tan\delta =G^{\prime \prime }/G^{\prime }$; $G^{\prime \prime }$, loss modulus; $G^{\prime }$, storage modulus) of the emulsions stabilized by OSA-PGS. Oil volume fraction: (**d**) 0.05, (**e**) 0.10, and (**f**) 0.20. Data were obtained by oscillation frequency sweep test. Lines indicate tanδ = 1.

Emulsion-filled gels can be produced with a wide range of rheological properties, which depend on various factors, such as the oil content, gelling agent content, and interactions among the components [22,23,37]. Changes in τ as a function of $\dot{\gamma }$ of emulsions stabilized by OSA-PGS are presented in (Figure 2). All of the emulsions displayed shear-thinning flow behavior, with τ increasing with increasing $\dot{\gamma }$ (or apparent viscosity [$\eta_{app}$] decreasing with increasing $\dot{\gamma }$) (Figure S2). This may be explained by shear-induced structural breakdown and elongation of the swollen starch granules in the shear direction. Small stresses at low $\dot{\gamma }$ were unable to disrupt the flocs. However, at sufficiently high $\dot{\gamma }$, the flocs were disrupted, which reduced the viscosity. This is similar to emulsions prepared in combination with gelling agents such as guar gum [38], zein [14], and whey protein isolate [6]. In chili sauces formulated with modified starches, pseudoplastic behavior reportedly promotes mixability and pumpability during processing, flowability when the sauce is poured from the bottle, and gel-like properties when the sauce is coated on foods. These are important factors in the design of semisolid food systems [19].

At the same OSA-PGS concentration, $\eta_{app}$ increased with increasing Φ (Figure S2). This was due to an increase in shear resistance due to increased particle–particle collisions in the gel-like matrix held together by interactions between the active filler particles and OSA-PGS. At the same Φ, as the OSA-PGS concentration increased, the $\eta_{app}$ of the emulsion increased because OSA-PGS acts as a thickener in the continuous phase (Figure S2). These results are generally consistent with those of emulsions thickened using polysaccharides, such as waxy maize and potato starches, as well as xanthan gums [39,40]. It is noteworthy that OSA-PGS had two major roles at the particle interface and in the continuous phase of the emulsions. Additionally, the maize starch used in this study contained approximately 30% amylose and has been reported to form inclusion complexes with lipids such as fatty acids, phospholipids, and monoacylglycerides [41,42]. Hence, intergranular networks composed of amylose-inclusion complexes with oil droplets increasingly affected the $\eta_{app}$ with increasing Φ and OSA-PGS concentrations. 

The Herschel–Bulkley model was used to describe the flow curves quantitatively, and the three associated parameters of $\tau_{0}$, n, and K are listed in Table 1. $\tau_{0}$ indicates the amount of stress that the fluid may experience before it yields and begins to flow; n indicates the degree to which the fluid is shear-thinning or shear-thickening; and K is a simple constant of proportionality. The coefficient of determination (R2) and *p* values for fitting to the Herschel–Bulkley model were >0.99 and <0.0001, respectively, indicating that the model fit the experimental data well. The Herschel–Bulkley model was used because it incorporates the Newton, Bingham, and Ostwald–de Waele models and has been widely applied to describe the rheological properties of food products. Since the K values of the Herschel–Bulkley model have different units from each other, a modified power law model ($\tau =\tau_{1}\times {\left(\dot{\gamma }/1{\;s}^{–1}\right)}^{n^{\prime }}$; $\tau_{1}$, stress at 1 s^–1^; $n^{\prime }$, modified flow index) was additionally used to fit the flow curve, and the two associated parameters are also listed in Table 1 [43]. The R2 and *p* values for fitting to the modified power law model were >0.98 and <0.0001, respectively, indicating the model also fit the flow data well. ANOVA showed that Φ and OSA-PGS concentrations significantly affected the Herschel–Bulkley model parameters and the modified power law model parameters (*p* < 0.05).

Yield stress is generally expected in emulsion-filled gels because it imparts stability in low-stress situations (<$\tau_{0}$), such as during storage and transportation. The higher the value of $\tau_{0}$, the lower the likelihood of structural changes that lead to instability. In this study, emulsions with Φ = 0.20 or 10% *w*/*v* OSA-PGS had high $\tau_{0}$ and were categorized as Herschel–Bulkley fluids (Table 1). At Φ = 0.20, $\tau_{0}$ increased with increasing OSA-PGS concentration, because OSA-PGS in the continuous phase changed the emulsion system to a gel-like structure. $\tau_{0}$ increased with increasing Φ because a larger number of active fillers contributed to increased resistance to shear and reinforcement of the structure in the continuous phase. $\tau_{1}$ was also influenced by Φ and OSA-PGS concentration in the same trend as $\tau_{0}$. The consistency index of emulsions increased with an increase in OSA-PGS concentration because the consistency index signifies the viscous nature of the system. The value of n and $n^{\prime }$ was <1 for all emulsions, indicating that the emulsions were shear-thinning. Since $n^{\prime }$ = 0 for solid and $n^{\prime }$ = 1 for Newtonian fluid [44], the value of $n^{\prime }$ decreased as Φ or OSA-PGS concentration increased.

Dynamic oscillatory shear tests are typically used to determine the viscoelastic properties of food systems. The $G^{\prime }$ and $G^{\prime \prime }$ moduli of emulsions stabilized by OSA-PGS are shown in Figure S3. They were essentially OSA-PGS concentration and frequency dependent across the range of frequencies tested. Regardless of Φ, both moduli showed an increasing trend with increasing oscillatory frequency or OSA-PGS concentration. A crossover frequency, where $G^{\prime }$ = $G^{\prime \prime }$, was observed in the emulsions with 3% *w*/*v* OSA-PGS and Φ = 0.05–0.10. This behavior indicated typical viscoelastic properties. At the same OSA-PGS concentration, $G^{\prime }$ showed that the emulsified oil droplets acted as strongly active filler particles and hence dramatically enhanced the gel strength.

The loss tangent descriptor was used to compare the viscoelastic characteristics of the examined emulsions (Figure 2). In both sets of samples prepared with 3–5% *w*/*v* OSA-PGS at Φ = 0.05–0.10, a decline in the tanδ value was observed in the range of tested frequencies. Emulsions made with 10% *w*/*v* OSA-PGS had nearly constant (<1) tanδ values across the entire range of frequencies applied, suggesting a more rigid network. This finding shows that these systems had a weak gel-like structure (0.1 < tanδ < 1), which is typical of dressings and mayonnaises. Similar results have been reported for other emulsion-filled gels thickened with gelling agents [11,45]. Moreover, emulsions with greater Φ had lower tanδ values at the same OSA-PGS concentration (Figure 2). This indicated that the oil droplets acted as space fillers in the gels, and the interaction between OSA-PGS covering the droplets and OSA-PGS in the aqueous phase generated a more elastic gel matrix. These rheological properties mean that oil droplets act as active fillers in the emulsion-filled gel systems, because OSA-PGS acts as both an emulsifier and a gelling agent.

### 3.3. Droplet Diameter and Storage Stability of the Emulsions

**Figure 3:**
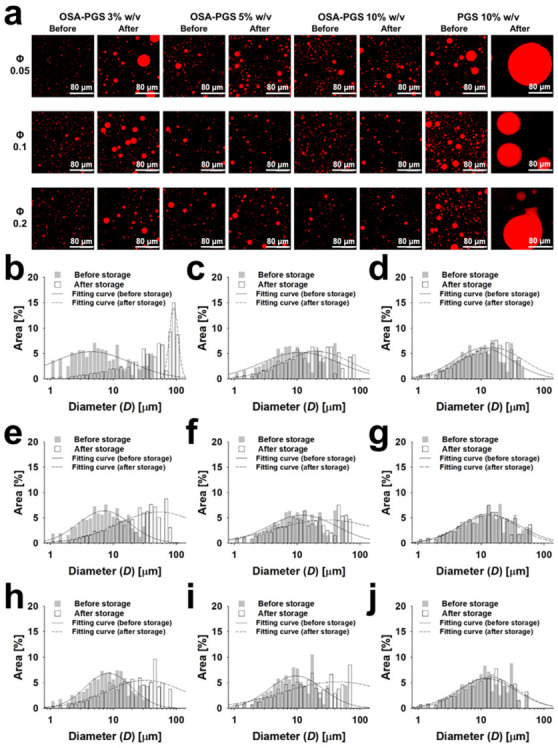
(**a**) Confocal laser scanning micrographs of emulsions stabilized by pregelatinized maize starch (PGS) or PGS modified with octenyl succinic anhydride (OSA-PGS) (Φ, oil volume fraction), before and after storage for 30 days. (**b**–**j**) Changes in the droplet diameter distribution of emulsions stabilized by OSA-PGS before and after storage for 30 days. OSA-PGS concentration: (**b**,**e**,**g**) 3, (**c**,**f**,**i**) 5, and (**d**,**g**,**j**) 10% *w/v*; oil volume fraction: (**b**–**d**) 0.05, (**e**–**g**) 0.10, and (**h**–**j**) 0.20. Data were obtained from confocal laser fluorescence micrographs of emulsions using image J image-processing software. Lines indicate the fitted curves based on the equation $\mathrm{Area}\;\left[\%\right]=ae^{-0.5{\left(\ln\left(D/D_{0}\right)/b\right)}^{2}}$; a and b, constants; $D_{0}$, D at the peak.

The microstructure of emulsions stored for 1 or 30 days under ambient conditions was observed by CLSM (Figure 3). Emulsions containing 3% *w*/*v* OSA-PGS at any of the three Φ conditions showed definite differences in droplet size between the 1 and 30 days of storage. By contrast, the samples containing 5–10% *w*/*v* OSA-PGS showed barely any change in droplet size during 30 days of storage. High coalescence occurred after 30 days of storage when the emulsion was stabilized using 10% *w*/*v* PGS. This suggests that OSA was responsible for PGS adsorption and stabilization at the oil/water interface. The droplet diameters were measured on the CLSM images using ImageJ, and the droplet diameter distribution was evaluated using Mastersizer 2000 software (Malvern, UK; Figure 3). As in the CLSM images, the oil droplets with 3% *w*/*v* OSA-PGS showed the greatest change in diameter. The oil droplets with 5% *w*/*v* OSA-PGS showed a slight change in size, whereas those with 10% *w*/*v* OSA-PGS did not change in size throughout the 30 days of storage.

The mean droplet diameters ($D_{1,0}$, $D_{3,2}$, and $D_{4,3}$) of the emulsions stabilized solely by 3–10% *w*/*v* OSA-PGS, with different Φ values (0.05–0.20), are shown in Table 2. One day after emulsion preparation, the mean droplet diameter was on the order of micrometers. The *D*_3,2_ of the droplets increased with increasing Φ at the same OSA-PGS concentration and with increasing OSA-PGS concentration at the same Φ. This may be due to the fixed volume of the emulsions (50 mL). When the OSA-PGS concentration was increased at a constant Φ, the viscosity of the aqueous phase increased, possibly causing the formation of larger droplets.

The stability of the emulsions was assessed by measuring the change in mean droplet diameter ($D_{1,0}$, $D_{3,2}$, and $D_{4,3}$) after 30 days of storage (Table 2). There was a change in the $D_{1,0}$ of the emulsions stabilized by 3% *w*/*v* OSA-PGS with Φ = 0.05–0.20 after 30 days of storage. Additionally, there was a significant change in the $D_{1,0}$ of the emulsions with Φ = 0.05 at an OSA-PGS concentration of 5% *w*/*v* after 30 days of storage. Four samples displayed significant changes in $D_{1,0}$ after 30 days, and these had the lowest $\tau_{0}$ values (Table 1). Samples with a relatively high fluidity and low $\tau_{0}$, prepared with 3–5% *w*/*v* OSA-PGS with Φ = 0.05–0.10, showed a significant increase in droplet size due to droplet coalescence during 30 days of storage. By contrast, the $D_{1,0}$ of the emulsions prepared with 5–10% *w*/*v* OSA-PGS with Φ = 0.10–0.20 did not change significantly after 30 days of storage.

A significant change was observed in the $D_{3,2}$ and $D_{4,3}$ of the emulsions stabilized with 3% *w*/*v* OSA-PGS (Table 2). $D_{4,3}$ is more sensitive to large oil droplets than $D_{3,2}$ and $D_{1,0}$. A few droplets with a diameter > 5 μm after 30 days of storage, as indicated by a high $D_{4,3}$ value, were observed in the emulsion stabilized with 3% *w*/*v* OSA-PGS under all three Φ conditions. This was likely due to an insufficient amount of OSA-PGS to cover the droplets and to the lower viscoelastic properties of the continuous phase. By contrast, the emulsions stabilized with 10% *w*/*v* OSA-PGS under all three Φ conditions showed no significant change in $D_{4,3}$ after 30 days of storage. This suggests that the emulsion-filled gels remained stable at ambient temperature with no droplet coalescence.

## 4. Conclusions

Pregelatinized maize starch modified with OSA can be used as both an emulsifier and gelling agent to prepare emulsion-filled gels in the absence of additional emulsifiers, gelling agents, or heat treatment. Confocal images showed that the application of OSA-PGS stabilized the emulsion, indicated by the formation of a thick layer surrounding the surface of the oil droplets and gelled the aqueous phase simultaneously. Thus, in these emulsion-filled gel systems, oil droplets acted as active fillers. All of the emulsions produced with 3–10% *w*/*v* OSA-PGS and Φ = 0.05–0.20 exhibited shear-thinning flow behavior, and only those with 10% *w*/*v* OSA-PGS were categorized as Herschel–Bulkley fluids. Increasing the OSA-PGS concentration increased the values of the $G^{\prime }$ and $G^{\prime \prime }$ moduli but decreased the value of the tanδ. Emulsions containing 3% *w*/*v* OSA-PGS had a liquid character ($G^{\prime \prime }$ > $G^{\prime }$), whereas those composed of 5–10% *w*/*v* OSA-PGS demonstrated structural formation ($G^{\prime }$ > $G^{\prime \prime }$). Gelation of the emulsions prepared with Φ = 0.20 or 10% *w/v* OSA-PGS was observed. The change in droplet size during 30 days of storage was used as an indicator of the physical stability of the emulsions. The use of 10% *w*/*v* OSA-PGS in dispersions with Φ = 0.05–0.20 generated stable emulsions, in which the mean droplet diameter ($D_{1,0}$, $D_{3,2}$, and $D_{4,3}$) was maintained during 30 days of storage under ambient conditions.

Conventional emulsion-filled gels are manufactured via a complicated and cumbersome process. This study, which simplified this complex process, has important practical and cost implications for situations when emulsion-filled gels are stabilized solely using starch-derived materials. These results provide the basis for a rational emulsion-filled starch gel design and demonstrate that the rheological behavior of these materials is governed by OSA-PGS acting as both an emulsifier and a gelling agent and by oil droplets acting as active fillers. This suggests the possibility of a diverse applications of these emulsion-filled starch gels within the food industry, including use as fat-replacement ingredients and in encapsulation systems for the delivery of nutraceuticals.

## Figures and Tables

**Table 1:** Parameters ($\tau_{0}$, yield stress; K, consistency index; n, flow index) obtained Herschel–Bulkley model fitted curves ($\tau =\tau_{0}+K{\dot{\gamma }}^{n}$; τ, shear stress; $\dot{\gamma }$, shear rate) and parameters ($\tau_{1}$, stress at 1 s^–^^1^; $n^{\prime }$, modified flow index) obtained from modified power law model fitted curves ($\tau =\tau_{1}\times {\left(\dot{\gamma }/1\;s^{–1}\right)}^{n^{\prime }}$ ) of the flow behavior plots (τ versus $\dot{\gamma }$ ) for the oil-in-water emulsions stabilized by pregelatinized maize starch modified with octenyl succinic anhydride (OSA-PGS) ^a^.

Oil Volume Fraction	OSA-PGS Concentration (% *w*/*v*)	$\tau_{0}\;(\mathrm{Pa})$	K	n	$\tau_{1}\;(\mathrm{Pa})$	$n^{\prime }$
0.05	3	-	0.3 ± 0.0 a	0.7 ± 0.0 d	0.3 ± 0.0 a	0.67 ± 0.00 f
	5	-	1.1 ± 0.0 bc	0.7 ± 0.0 d	1.1 ± 0.0 a	0.63 ± 0.00 e
	10	18.4 ± 0.9 c	4.6 ± 0.2 e	0.6 ± 0.0 ab	8.5 ± 0.1 c	0.57 ± 0.00 c
0.10	3	-	0.4 ± 0.0 a	0.7 ± 0.0 d	0.4 ± 0.0 a	0.66 ± 0.00 f
	5	-	1.2 ± 0.0 c	0.6 ± 0.0 c	1.2 ± 0.0 a	0.63 ± 0.00 e
	10	53.9 ± 1.5 d	2.5 ± 0.1 d	0.8 ± 0.0 e	11 ± 0.2 d	0.54 ± 0.00 b
0.20	3	0.6 ± 0.0 a	0.8 ± 0.0 b	0.6 ± 0.0 bc	0.9 ± 0.0 a	0.62 ± 0.00 d
	5	5.7 ± 0.3 b	2.5 ± 0.1 d	0.6 ± 0.0 a	3.7 ± 0.0 b	0.57 ± 0.00 c
	10	171.2 ± 3.1 e	5.4 ± 0.3 f	0.8 ± 0.0 e	40 ± 0.9 e	0.48 ± 0.00 a

^a^ Within a column, values followed by different letters (a to f) are significantly different (*p* < 0.05).

**Table 2:** Mean diameters (number mean.diameter, $D_{1,0}$; Sauter mean diameter, $D_{3,2}$; de Broukere mean diameter, $D_{4,3}$ ) of emulsions stabilized by pregelatinized maize starch modified with octenyl succinic anhydride (OSA-PGS) before and after storage for 30 days at 25 °C and pH 7 ^a^.

Oil Volume Fraction	OSA-PGS Concentration % (*w*/*v*)	$D_{1,0}\;(\mu m)$		$D_{3,2}\;(\mu m)$		$D_{4,3}\;(\mu m)$	
		Before Storage	After Storage	Before Storage	After Storage	Before Storage	After Storage
0.05	3	2.5 ± 0.1 Aa	4.8 ± 0.3 Cb	10.1 ± 3.1 Aa	55.0 ± 7.8 Db	19.5 ± 9.7 Aa	78.3 ± 12.2 Cb
	5	3.4 ± 0.1 ABa	4.4 ± 0.3 ABCb	14.8 ± 1.7 ABa	24.7 ± 5.0 ABCb	26.6 ± 2.8 Aa	42.6 ± 11.1 ABa
	10	3.8 ± 0.1 Ba	4.0 ± 0.2 ABCa	14.1 ± 2.1 ABa	16.0 ± 1.3 Aa	22.8 ± 4.8 Aa	23.3 ± 1.8 Aa
0.10	3	3.1 ± 0.2 ABa	4.6 ± 0.2 BCb	9.7 ± 1.9 Aa	30.9 ± 3.7 Cb	14.6 ± 3.9 Aa	47.6 ± 4.8 Bb
	5	3.7 ± 0.7 Ba	3.8 ± 0.6 Aa	15.2 ± 2.3 ABa	24.2 ± 1.5 ABCb	26.9 ± 8.7 Aa	42.3 ± 7.1 ABa
	10	3.8 ± 0.4 Ba	3.9 ± 0.1 ABa	18.8 ± 3.1 Ba	18.3 ± 1.0 ABa	31.6 ± 6.2 Aa	31.9 ± 4.4 ABa
0.20	3	3.4 ± 0.1 ABa	4.5 ± 0.2 ABCb	11.1 ± 1.1 Aa	27.4 ± 3.1 BCb	17.3 ± 2.5 Aa	43.3 ± 6.2 ABb
	5	3.7 ± 0.1 Ba	4.0 ± 0.2 ABCa	13.3 ± 2.8 ABa	24.0 ± 2.3 ABCb	23.5 ± 11.1 Aa	42.2 ± 4.6 ABa
	10	3.9 ± 0.3 Ba	4.0 ± 0.2 ABCa	16.2 ± 3.8 ABa	16.3 ± 3.3 Aa	25.1 ± 8.1 Aa	25.0 ± 7.9 Aa

^a^ Within columns, values followed by different letters (A, B, and C) are significantly different (*p <* 0.05). Within rows, values followed by different letters (a and b) are significantly different (*p <* 0.05).

## Data Availability

Not applicable.

## Supplementary Materials

The following are available online at https://www.mdpi.com/article/10.3390/foods10040837/s1, Figure S1: appearance of emulsions stabilized by OSA-PGS, Figure S2: apparent viscosity of emulsions stabilized by OSA-PGS, and Figure S3: loss and storage moduli of emulsions stabilized by OSA-PGS.
