# Supplementary material for: Rheology, Microstructure, and Storage Stability of Emulsion-Filled Gels Stabilized Solely by Maize Starch Modified with Octenyl Succinylation and Pregelatinization
Source: Foods. 2021 Apr 12;10(4):837. doi: 10.3390/foods10040837 (PMC8069085; doi:10.3390/foods10040837)
Supplement: Supplementary file 1 [file foods-10-00837-s001.pdf]

foods

Rheology, microstructure, and storage stability of emulsion-filled gels stabilized solely by maize starch modified with octenyl succinylation and pregelatinization

Myeongsu Jo[1,2,†], Min Jea Chang[4,†], Kelvin K. T. Goh[5], Choongjin Ban[6,] and Young Jin Choi[1,2,3,*]**

[1] Department of Agricultural Biotechnology, Seoul National University, Seoul 08826, Republic of Korea; cmso5517@snu.ac.kr (M.J.)
[2] Center for Food and Bioconvergence, Seoul National University, Seoul 08826, Republic of Korea
[3] Research Institute of Agriculture and Life Sciences, Seoul National University, Seoul 08826, Republic of Korea
[4] Dongsuh Foods Corporation, Incheon 21314, Republic of Korea; mjjang@dongsuh.co.kr (M.J.C.)
[5] School of Food and Advanced Technology, Massey University, Palmerston North 4442, New Zealand; K.T.Goh@massey.ac.nz (K.K.T.G.)
[6] Department of Environmental Horticulture, University of Seoul, Seoul 02504, Republic of Korea
* Correspondence: choiyj@snu.ac.kr (Y.J.C.); pahncj@uos.ac.kr (C.B.); Tel.: +82 2 880 4851 (Y.J.C.)
† Co-first authors, these authors contributed equally to this work

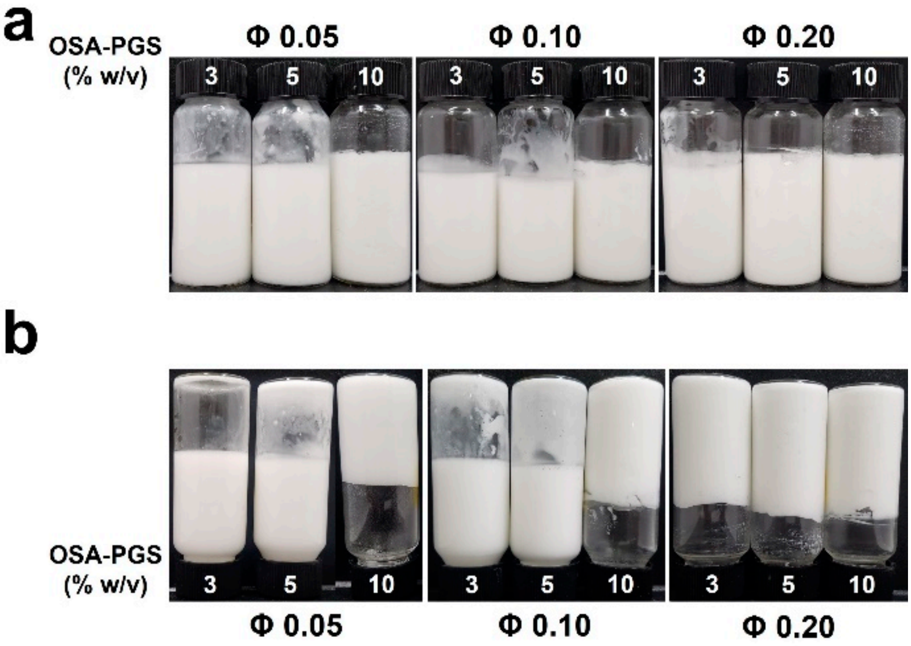

Figure S1. Appearance of the emulsions stabilized by 3, 5, and 10% *w/v* pregelatinized maize starch modified with octenyl succinic anhydride (OSA-PGS) (Φ, oil volume fraction). Side views of vials placed (**a**) upright and (**b**) inverted after the emulsion preparation.

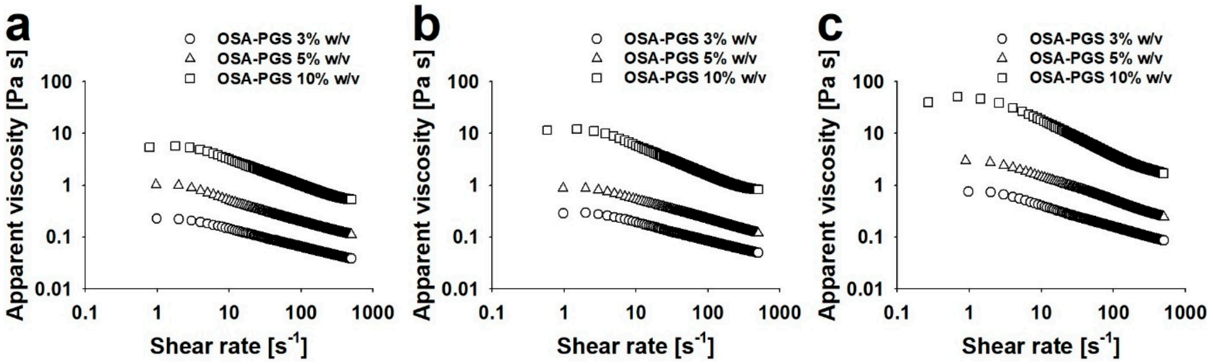

Figure S2. Apparent viscosity of emulsions stabilized by pregelatinized maize starch modified with octenyl succinic anhydride (OSA-PGS). Oil volume fraction: (**a**) 0.05, (**b**) 0.10, and (**c**) 0.20. Data were obtained from flow behavior plots of shear stress *vs*. shear rate.

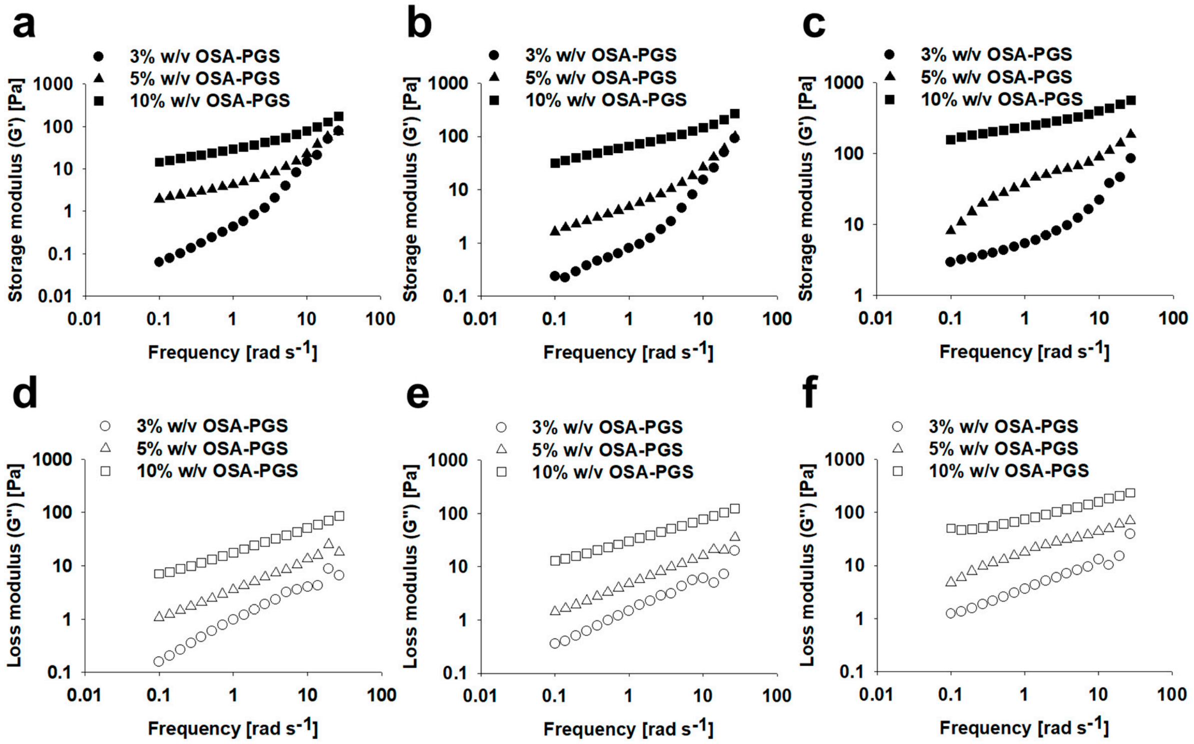

Figure S3. Loss (G^') and storage (G^'') moduli of emulsions stabilized by pregelatinized maize starch modified with octenyl succinic anhydride (OSA-PGS). Oil volume fraction: (**a** and **d**) 0.05, (**b** and **e**) 0.10, and (**c** and **f**) 0.20. Data were obtained by oscillation frequency sweep test.
